# ReplicationDomain: a visualization tool and comparative database for genome-wide replication timing data

**DOI:** 10.1186/1471-2105-9-530

**Published:** 2008-12-10

**Authors:** Nodin Weddington, Alexander Stuy, Ichiro Hiratani, Tyrone Ryba, Tomoki Yokochi, David M Gilbert

**Affiliations:** 1 Department of Biological Sciences, Florida State University, Tallahassee, Florida 32306, USA

## Abstract

**Background:**

Eukaryotic DNA replication is regulated at the level of large chromosomal domains (0.5–5 megabases in mammals) within which replicons are activated relatively synchronously. These domains replicate in a specific temporal order during S-phase and our genome-wide analyses of replication timing have demonstrated that this temporal order of domain replication is a stable property of specific cell types.

**Results:**

We have developed ReplicationDomain  as a web-based database for analysis of genome-wide replication timing maps (replication profiles) from various cell lines and species. This database also provides comparative information of transcriptional expression and is configured to display any genome-wide property (for instance, ChIP-Chip or ChIP-Seq data) via an interactive web interface. Our published microarray data sets are publicly available. Users may graphically display these data sets for a selected genomic region and download the data displayed as text files, or alternatively, download complete genome-wide data sets. Furthermore, we have implemented a user registration system that allows registered users to upload their own data sets. Upon uploading, registered users may choose to: (1) view their data sets privately without sharing; (2) share with other registered users; or (3) make their published or "in press" data sets publicly available, which can fulfill journal and funding agencies' requirements for data sharing.

**Conclusion:**

ReplicationDomain is a novel and powerful tool to facilitate the comparative visualization of replication timing in various cell types as well as other genome-wide chromatin features and is considerably faster and more convenient than existing browsers when viewing multi-megabase segments of chromosomes. Furthermore, the data upload function with the option of private viewing or sharing of data sets between registered users should be a valuable resource for the scientific community.

## Background

In eukaryotic cells, segments of chromosomes replicate via the synchronous firing of clusters of replication origins [1]. These segments or "replication domains" replicate in a defined temporal order during S-phase. This replication-timing program is cell type specific [2], and developmentally regulated changes in this program are associated with changes in chromatin structure and gene expression [2-5]. In particular, a global re-organization of this replication-timing program occurs during the differentiation of mouse embryonic stem cells (mESCs), with changes occurring at the level of large (~600 kb) chromosomal domains reflecting global re-positioning of sequences within the nucleus [2]. Moreover, pluripotent cells can be distinguished from differentiated cells not only by differences in their replication timing profiles but by their smaller and more numerous replication domains [2]. Hence, replication timing is a unique epigenetic property of chromatin in that it is regulated at the level of megabase-sized domains. Establishing replication maps for various tissues is likely to provide a database of chromosome segments that undergo large changes in organization during differentiation.

The significance of a replication-timing program has remained elusive. In several model systems, defects in replication-timing are associated with defects in chromosome condensation, sister chromatid cohesion, and genome stability [6,7]. Abnormal replication-timing control has become a clinical marker for predicting malignant cancers [8-12]. In particular, specific chromosome translocations result in a chromosome-wide delay in replication timing that triggers additional chromosome translocations at a high frequency [13,14]. Cells from patients with several inherited human diseases show defects in replication-timing that correlate with mis-regulation of genes during development [15-18]. Also, replication domains are separated by timing transition regions (the domain boundaries) that appear to be devoid of replication origins, requiring that a single replication fork travel very long distances between early and late replicating domains [2,19,20]. Evidence suggests that genes lying within these transition regions are prone to DNA damage [21,22]. While very few such boundaries have been mapped, their cell-type specificity suggests the possibility that differential organization of replication domains may contribute to cell type specific predispositions to certain types of DNA damage. Hence, establishing a database of replication timing profiles for various tissues and their relationship to transcription and other chromosomal properties is a prerequisite for understanding the roles of replication timing in chromosome-based diseases. These roles may extend beyond epigenetic regulation of transcription: the locations and directions of replication forks, the organization of replication complexes that coordinate replication of large domains, and the locations of domain boundaries may constitute an epigenetic basis for tissue-specific or cancer-promoting differences in genome stability.

Surprisingly few genome-wide studies of replication timing have been performed [23]. Early studies in *Drosophila *cells with cDNA arrays [24], or in human cells using BAC arrays [25] did not provide the resolution to define replication domains and their boundaries. A tiling array study of human ENCODE regions covering 1% of the genome was also not able to precisely delineate replication domains, likely because they are typically larger than the 500 kb segments queried by this study [26,27]. Other high-resolution studies of specific chromosome segments in human and *Drosophila *[28-30] also did not delineate domain structure but noted that the relationship between replication timing and transcription was best described at the level of large multi-genic regions rather than individual genes [30].

We have recently mapped replication domain structure genome-wide in mouse embryonic stem cells (mESCs) and their differentiated counterparts [2]. We recognized the need for a comprehensive database to display these profiles and compare them between cell lines as well as to other chromosome-wide properties such as transcriptional activity or other epigenetic marks. While this can in principle be done on other public web browsers such as the UCSC Genome Brower , in many cases it is desirable to quickly visualize one's data from individual replicates or using unpublished data in a format that is not appropriate for public viewing. Such comprehensive public databases are becoming complex with the number of tissue and cell-type specific data sets that the reader must browse through. Generally speaking, they are tailored toward the display of static features of chromosomes, rather than dynamic cell-type specific features. Moreover, because of the increasing complexity of genome browsers, viewing large chromosomal regions necessary to visualize replication domains tends to be very slow (e.g. a 5-Mb chromosome segment takes several tens of seconds to display on the UCSC Genome Browser, but 2–3 seconds on ReplicationDomain).

For these reasons, we developed ReplicationDomain as a centralized repository that enables rapid comparative analysis of the genomic landscape for replication domain organization, with the potential to compare these properties to any other genome-wide chromosome data sets, such as those from ChIP-Chip or ChIP-Seq experiments. Our published microarray data sets are publicly available for any non-registered user to view and download. Furthermore, we have implemented a user registration system that allows registered users to upload their own data sets. Users have three options for data security, either to view their data sets privately without sharing (Über Private), share with other registered users (Private), or make their data sets publicly available on condition that they are published or "in press" in peer-reviewed journals (Public). In the future, we plan to implement additional data security mechanisms that will allow sharing of data sets only with designated registered users. The ability of registered users to upload data sets for private viewing facilitates confidential sharing of data prior to publication, or for quality control checks. ReplicationDomain uses an interactive interface designed to be intuitive for users familiar with the UCSC Genome Browser, with unique features optimized for rapid viewing of multi-megabase domain-wide chromosome properties and the option to jump to the same region of interest in the UCSC Genome Browser. Our recent demonstration of global re-organization of replication domains during ESC differentiation [2] predicts that replication profiling will provide a rapid and comprehensive means to evaluate cell-type specific features of global genome organization. ReplicationDomain will provide a valuable resource to consolidate replication profiles, making them more accessible to view and identify cell-type specific properties. We encourage users to begin uploading data sets and suggesting features that will improve this database.

## Construction and content

### Current Contents

**Figure 1:**
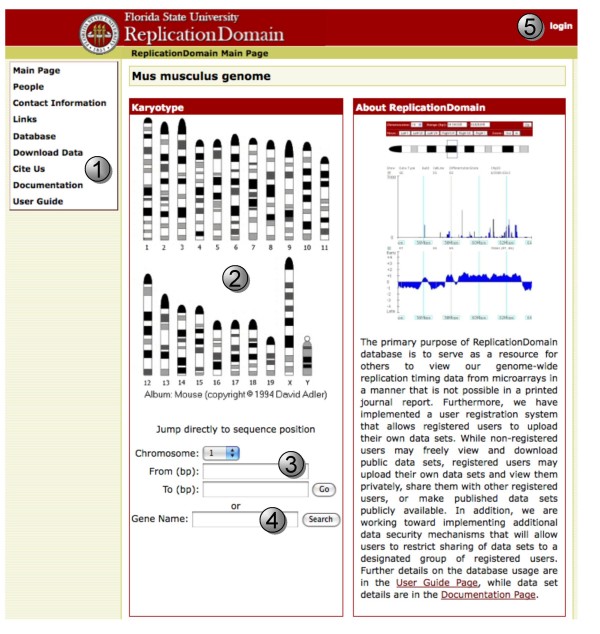
**ReplicationDomain Main Page**. Buttons correspond to descriptions in the text.

Currently, ReplicationDomain accommodates 10 microarray data sets publicly available without a login requirement. They consist of 7 replication profiles (genome-wide replication timing data) with probes spaced every 5.8 kb for three different mESC lines (D3, TT2 and 46C) either prior to differentiation (indicated ESC) or after differentiation to neural precursor cells (NPCs) either using defined medium in monolayer culture (indicated NPC/ASd6) or in conditioned medium as embryoid bodies (indicated NPC/EBM9), as well as one data set for iPS (induced pluripotent stem) cells. One replication timing profile has been performed with probes spaced every 100 bp along regions of chromosome 6 and 7 for undifferentiated mESC line D3. In addition, there are 2 transcription profiles for mESC line D3 and its NPC/EBM9 differentiated counterpart. The details of how these data sets were collected are explained under "Documentation" (Figure 1, button 1). Further details and conclusions derived from these data sets are presented in Hiratani et al [2]. Briefly, replication-timing data were obtained by hybridizing early and late replication intermediates simultaneously to Nimblegen oligonucleotide arrays containing evenly spaced probes (CGH arrays). The Data Display page shows the log_2 _ratio of early to late replicating intermediates for each probe as a function of its position along the length of any given chromosome. Transcription data were obtained from Affymetrix arrays, using standard methods described under Documentation (Figure 1, button 1) or in Hiratani et al [2]. What is displayed is a bar, the height of which is the relative signal intensity, and the width of which delineates the start and stop sites of transcription. Gold-colored bars represent genes deemed sufficiently above background to be called "Present" (i.e. expressed) while garnet-colored bars represent genes considered to be "Absent" (i.e. silent) or very lowly expressed. The web site is designed to anticipate any tab-delimited text data file as long as each record indicates the chromosome, start and end nucleotide coordinates, and a data value. Importantly, as long as the file structure follows this format, the data values can derive from any other genome-wide application such as ChIP-Chip, ChIP-Seq, and RNA-Seq.

### System Architecture and Implementation

#### Software and Hardware

**Figure 2:**
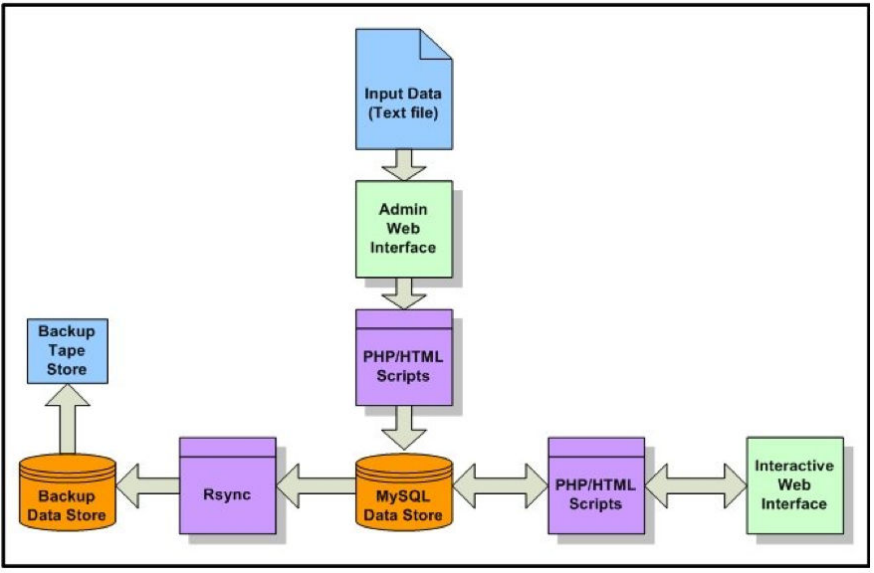
**An Overview of ReplicationDomain Data Flow**. Input data as text files is uploaded via administrative web interface, checked for errors, and inserted into MySQL database. Data is retrieved by interactive web interface for display. Data is archived via rsync to additional data store, data is written to tape from additional data store.

The ReplicationDomain software is an integrated system consisting of a relational database, PHP scripts, administrative interface, and an interactive web-based user interface (Figure 2). Upon upload as tab-delimited text, each data set is parsed and inserted into the database, which can then be queried for data in particular chromosomes, regions, or genes. PHP and HTML scripts are used to connect the user interface and uploaded data sets to the database, which is backed up twice daily to separate hard disk and weekly to tape. The database is hosted on MySQL Server 5.0.51, a multithreaded relational database management system. The Web based user interface, internal administrative interface, administrative scripts, and website statistics are programmed using Apache/2.2.9 (FreeBSD), mod_ssl 2.2.9, OpenSSL 0.9.8e DAV/2, and PHP 5.2.6 with Suhosin-Patch running on a Free BSD version 7.0 64-bit operating system. Graphical output is generated with ImageMagick 6.4.1.8 and GraphicsMagick 1.1.12, and access logs are recorded and analyzed using custom PHP scripts and Analog 6.0.1.

## Utility and discussion

ReplicationDomain is designed to quickly and conveniently examine and compare properties of chromosomes that are important for their higher order structure and function, particularly as it relates to the organization of replication timing domains. It functions similar to other genome browsers, but has unique features that allow one to examine and compare microarray data for replication timing to steady state transcript levels (or any other genome-wide feature) with ease. Data can be downloaded as a tab-delimited text file or saved as a JPEG file for figure assembly. We also provide a link to the UCSC Genome Browser for quick access to additional data for the chromosomal region of interest. Members of the Gilbert lab utilize this site regularly as a tool to mine patterns in the genome indicative of functional changes in chromosome structure during differentiation (see "Downloading data for personal use"). Furthermore, we have implemented a user registration system that allows registered users to upload their own data sets, the details of which can be found under "Creating a ReplicationDomain account" and "Uploading your own data set" below. Regarding unpublished data sets, registered users can either view them privately without sharing or share with other registered users. For published or "in press" data sets, investigators have an option to release them for public access. We can accommodate data sets from any species with a physical genome map, but may need some time to assemble a new navigation path for each new species.

### Database Interface

ReplicationDomain is freely accessible at . It provides a novel tool to examine and compare microarray data for replication timing to other chromosomal properties. Different types of data sets can be aligned, by virtue of the dynamically generated graphical output. This unique feature allows investigators to compare replication timing and other chromosomal properties at any region of the genome under different developmental, genetic (e.g. gene knockout cell lines) and/or experimental conditions. Non-registered users may freely view and download public data sets. Registered users with a ReplicationDomain account may upload their own data sets and view them privately or share them with other registered users. Published or "in press" data sets can be added to the series of data sets public available. Further details of these procedures can be found below.

#### Getting to a region of interest

**Figure 3:**
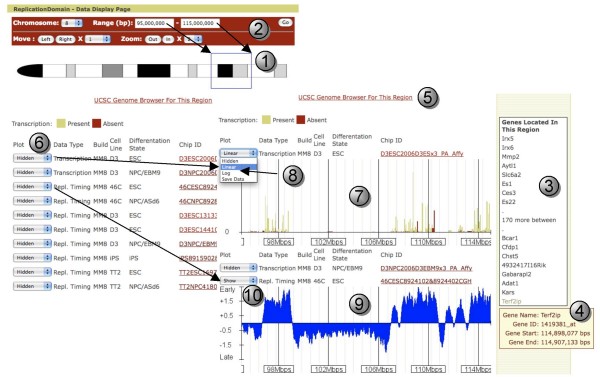
**Data Display Page: An Exemplary View of A Region of Chromosome 8**. Buttons correspond to descriptions in the text.

In the near future, species other than the mouse (*Mus musculus*) will be available and the user will need to first choose a species and be redirected to the data available for that species, similar to the UCSC Genome Browser. At present, only mouse data are uploaded so the viewer opens to the mouse page directly. From the Main Page (Figure 1), you can jump to the Data Display page (Figure 3) and view your favorite chromosomal region in one of three ways:

1) Click on the chromosome that contains your region on the ideogram (Figure 1, button 2). After being directed to the Data Display page of the chromosome, drag a rectangle around the region of interest on that chromosome (Figure 3, button 1).

2) Jump directly to a position by choosing the chromosome from the dropdown and typing in the desired base pair (bp) coordinates (Figure 1, button 3 and Figure 3, button 2).

3) Search for a Gene Name near the site of interest (Figure 1, button 4). You may be asked to choose from similar-named genes, particularly if you have typed in a partial gene name. When you arrive to the chromosome position, your gene of interest will appear in the center of the image. You will likely need to zoom out to see the context of your gene. Your gene will remain at the center as you zoom.

When you select a region of the chromosome, the Data Display page will display a floating gene name box (Figure 3, button 3) in the right column that contains the names and map positions of all genes within the selected region. If there are more than 16 genes, the first and last 8 genes are shown, with the number of genes between them indicated. Pointing your mouse cursor at a gene name will open a hover box that will provide its start and stop positions (Figure 3, button 4). Further information about this genomic region or the structure of the genes contained within it can be found by clicking the UCSC Genome Browser link (Figure 3, button 5).

#### Choosing data sets to view

Data sets on the Data Display page are chosen for viewing much as they are with the UCSC Genome Browser. All data sets are "Hidden" as a default, and can be viewed by using the dropdown menus (Figure 3, button 6). Relative transcription levels (Figure 3, button 7) can be plotted with either a linear or log scale (Figure 3, button 8) while replication-timing data is always plotted as a log_2 _ratio (Figure 3, button 9) by choosing "Show" (Figure 3, button 10). When these options are selected, graphical display of the data set will show up automatically. The y-axes are adjusted to the highest and lowest values in the entire data set (e.g. for transcription, the height of the y-axis provides an indication of the most highly transcribed gene in that data set). Details of each data set can be found by clicking on their Chip ID on the Data Display page (Figure 3, Chip ID column) or in the "Database" link in the main menu window (Figure 1, button 1). Definitions of the terms used to describe the data sets can be found under the "Documentation" link in the main menu (Figure 1, button 1).

#### Moving around

You can move to the left or the right, jump to a specific nucleotide position, or zoom in or out (2–8 fold) using the buttons at the top of the Data Display page (Figure 3, button 2), similar to what is done with the UCSC Genome Browser. ReplicationDomain also allows you to grab regions of the chromosome using your mouse cursor; simply drag a rectangle around the chromosomal region of interest either on the chromosome ideogram (Figure 3, button 1) or on individual data sets (Figure 3, buttons 7 and 9) and you will jump to that region. To orient your position relative to genes in the area, you can: (1) use the floating gene name box in the right column (Figure 3, button 3); (2) use the link to the UCSC Genome Browser; or (3) download the corresponding "Transcription" data with all gene names and positions.

#### Downloading data for personal use

**Figure 4:**
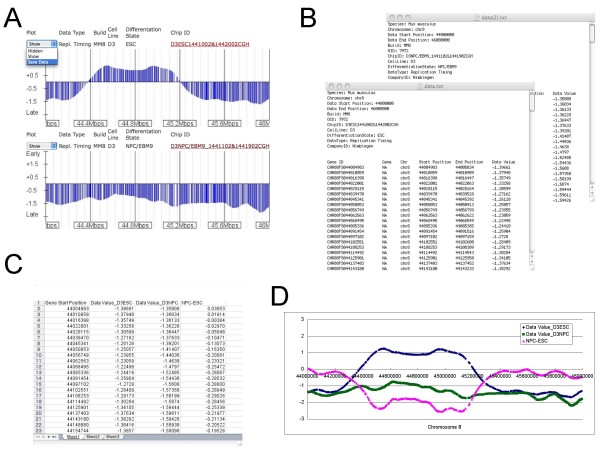
**An Exemplary Analysis Performed with ReplicationDomain**. Visual inspection with the aid of ReplicationDomain identifies a region where replication timing has changed during differentiation of ESCs to NPCs. Users can download the data shown on the screen by selecting the "Save Data" option of each data set (A). Downloaded data for each data set will be in a tab-delimited text file with a description at the top (B). Data from multiple differentiation states can be assembled and their differentials calculated in Microsoft Excel (C) and visualized graphically (D). The graph (D) shows a comparison of D3ESC (dark blue) and D3NPC/EBM9 (green) replication timing profiles and their differentials (NPC-ESC; magenta) on chr8:44,000,000–46,000,000.

For further analysis (an example is illustrated in Figure 4), you can download the data sets shown on your screen (Figure 4A). When this option is chosen, you will be prompted to select a location on your desktop for downloading all the data points for that data set contained within the selected genomic region. The data will be downloaded as a tab-delimited text file that will contain a description of the data at the top, followed by a table of data that you can move into any spreadsheet program of choice for further analysis (Figures 4B, 4C and 4D). You can download data representing a small region or up to an entire chromosome using this function. If you desire the complete data set for all chromosomes, that is provided separately through the "Download Data" link in the main menu window (Figure 1, button 1). Of course, you may also save any web page as a .pdf or take a snapshot using your own computer if you want to build quick figures for group presentations.

#### Comparing to other genome properties

At the top of the page is a link entitled "UCSC Genome Browser for this Region" (Figure 3, button 5). Clicking this button will open a new tab or browser page to the UCSC Genome Browser for the region viewed on ReplicationDomain. In addition, if you wish to upload your own data sets and compare to other data sets on ReplicationDomain, you will need to register yourself first (see "Creating a ReplicationDomain account" below for details). Registered users are entitled to upload their data sets as described below under "Uploading your own data set."

#### Creating a ReplicationDomain account

User registration is required for those who wish to upload their data sets or use the database interactively with other registered users. To create a ReplicationDomain account, please visit our ReplicationDomain Account Request page (a link is found on the User Guide page) and submit the Account Request Information form. A confirmation email will be sent to you with a ReplicationDomain username and a password, which will allow you to log in (Figure 1, button 5).

#### Uploading your own data set

**Figure 5:**
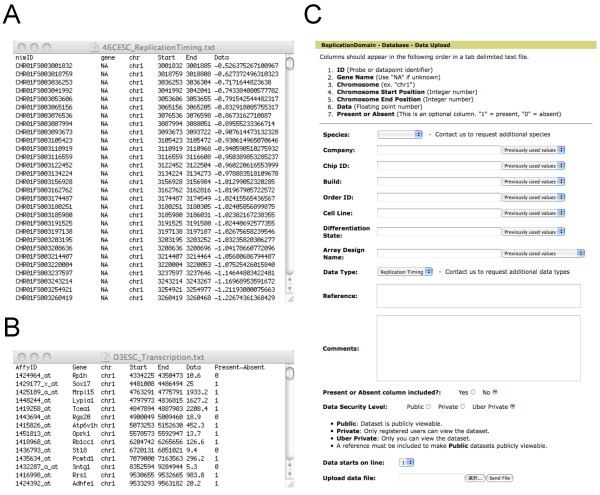
**Uploading Your Own Data Set**. (A, B) Data file structures. Exemplary tab-delimited text data files for replication timing (A) and transcription (B). (C) A screenshot of the "Data Upload" page.

To upload your data set, first generate a single, tab-delimited text file for each data set that includes the following 6 columns in this order as shown in Figures 5A and 5B: Gene/Probe ID (any unique identifier of genes or probes), Gene Name (type NA when unavailable), Chromosome, Chromosome Start, Chromosome End, and Data Value. You can also add a Present- Absent (P = 1, A = 0) column as a 7th column for transcription data sets. Then, log in with your ReplicationDomain account (Figure 1, button 5), go to the "Database" link from the main menu (Figure 1, button 1), click the "Upload data set" link and fill out the form (Figure 5C) with the requested attributes, select your text data file and hit "send." This should upload your data set, which will appear on the Data Display page for graphical display as well as on the list of data sets found under the "List my data sets" link on the Database page. All of the current 14 data set attributes are described under "*Definitions of Data Entry Terms*" below, which includes Data Security Level (description also on our Documentation page). Users can select Public, Private, or Über Private. Public data sets can be viewed by any user without a login requirement, but we require such data sets to be published or "in press" in peer-reviewed journals for database quality assurance purposes (i.e. a reference must be provided). Private data sets are viewable by all registered users with a ReplicationDomain account, while Über Private data sets are viewable only by the registered user who uploaded them. In the future, we anticipate implementing additional data security mechanisms that will allow sharing of data sets among designated registered users. Finally, existing data set attributes including Data Security Level can be edited later if necessary: click the "Database" link from the main menu, identify your data set from "List my data sets" link, hit "Edit," make necessary modifications, and hit "Update."

#### Definitions of Data Entry Terms

Data sets are defined by a combination of 14 entries, as described below (see also Figure 5C). Upon uploading data sets, registered users can either select terms from the dropdown list, or create a new term by filling in the blank.

##### Species

At present, we only have *Mus musculus*, but we intend to add *Homo sapiens *and *Drosophila melanogaster *soon. Contact us to create any new species page.

##### Company

Microarray product supplier name.

##### Chip ID

This is the unique identifier for each data set. While a "Chip ID" normally represents a single replicate experiment (e.g. one microarray hybridization), most data sets currently displayed on the Data Display page are averages of multiple replicates. Therefore, we have re-defined Chip ID as a string of characters combining individual "Chip ID" numbers and description of the data set identity. Chip ID is not useful except to communicate comments regarding a particular experiment. On the Data Display page, however, the Chip ID for each data set is set up as a link to the "Data Set Details" (Figure 3, Chip ID column; also accessible from the "Database" link on the main menu). The Chip ID is also useful for identifying data sets when downloading entire data sets through the "Download Data" link in the main menu.

##### Build

This indicates the version of the genomic sequence information that was used to assemble the microarray chip in the particular experiment (for example, mm7 or mm8 for the mouse). Builds change slightly as sequence information becomes updated, so the exact base pair position of any given DNA sequence will change as the sequence information becomes annotated. The build information indicated in each data set shows the build used for chromosomal coordinates of probes on the particular array type used.

##### Order ID

For Nimblegen data sets only.

##### Cell Line

This indicates the name of cell lines employed. At present, D3 [31], TT2 [32] AND 46C [33] are three different established mESCs.

##### Differentiation State

This is the tissue or tissue type represented by the cell line used and grown under the indicated conditions. Currently, there are four such differentiation states:

ESC: Undifferentiated ES cells

NPC/EBM9: The 9th day of differentiation following an established neural differentiation protocol that differentiates ESCs via embryoid bodies to Sox1 positive NPCs in conditioned medium [34]. NPC/ASd6: The 6th day of differentiation following an established neural differentiation protocol that differentiates ESCs to Sox1 positive NPCs in monolayer cultures using defined medium [33].

iPS: "Induced Pluripotent Stem Cells" re-programmed from tail-tip fibroblasts derived from a 129xBL-6 hybrid strain of mice to the pluripotent state as described [35].

##### Array Design Name

Microarray supplier and catalog number.

##### Data Type

Indicates the property being measured in the indicated experiment. At present, replication timing and transcription data are shown. In the future, data for other genome-wide properties of chromosomes may be displayed. Contact us to add new types of data such as ChIP-Chip or ChIP-Seq.

##### Reference

Data sets to be displayed publicly must include a reference.

##### Comments

We provide detailed microarray design information here but any additional comments can be added.

##### Present or Absent Column

For uploading transcription data sets that contain present-absent calls, specify here.

##### Data Security Level

Users can select Public, Private, or Über Private. Users can make their published or "in press" data sets publicly available by selecting "Public" and providing a reference under the entry term, *Reference*. Private data sets are viewable by all registered users with a ReplicationDomain account, while Über Private data sets are viewable only by the user who uploaded the data set.

##### Data Starts on Line

Usually starts on line 2, with line 1 being the column names.

## Conclusion

ReplicationDomain provides a user-friendly platform to view replication timing data from any organism and compare that data to other properties in a manner that is optimized for rapid viewing of multi-megabase segments of chromosomes. In addition to providing a consolidated and devoted database, ReplicationDomain provides the opportunity for researchers to share and analyze preliminary data sets with colleagues prior to providing public access. Although not as comprehensive as other databases, ReplicationDomain allows rapid linkage to the UCSC Genome Browser for cross-referencing to other databases. At present, the site contains only data sets collected in the Gilbert laboratory. In the future, we expect to serve as curators of a substantial database, and we expect ReplicationDomain to contain data from other laboratories and for other species, as well as other specific properties that are relevant to higher order domain structure of chromosomes. We invite others to use the web site and to create an account and upload their own data sets so that ReplicationDomain can be used to advance our understanding of the functional significance of a dynamic, developmentally regulated replication-timing program.

## Availability and requirements

ReplicationDomain is available at  for use by academic or non-academic users without restriction or charge.

## Authors' contributions

NW and AS are the web site designers, NW designed and programmed interactive web interface and updates/maintains administrative interface, IH generated the data and consulted in all aspects of the design, TR and TY assisted with implementation of the design and communications between biologists and computer scientists, AS designed hardware and software architecture, designed database structure, designed and programmed administrative interface and scripts, DMG conceived of the project and coordinated all aspects of its development. IH, AS, and DMG wrote the manuscript and all authors have read and approved the manuscript.

## References

[B1] Gilbert DM, Gasser SM, DePamphilis ML (2006). Nuclear Structure and DNA Replication. DNA replication and human disease.

[B2] Hiratani I, Ryba T, Itoh M, Yokochi T, Schwaiger M, Chang CW, Lyou Y, Townes TM, Schubeler D, Gilbert DM (2008). Global reorganization of replication domains during embryonic stem cell differentiation. PLoS Biol.

[B3] Gilbert DM (2002). Replication timing and transcriptional control: beyond cause and effect. Curr Opin Cell Biol.

[B4] Goren A, Cedar H (2003). Replicating by the clock. Nat Rev Mol Cell Biol.

[B5] Schwaiger M, Schubeler D (2006). A question of timing: emerging links between transcription and replication. Curr Opin Genet Dev.

[B6] Loupart M, Krause S, Heck MS (2000). Aberrant replication timing induces defective chromosome condensation in Drosophila ORC2 mutants. Curr Biol.

[B7] Pflumm MF, Botchan MR (2001). Orc mutants arrest in metaphase with abnormally condensed chromosomes. Development.

[B8] Amiel A, Elis A, Sherker S, Gaber E, Manor Y, Fejgin MD (2001). The influence of cytogenetic aberrations on gene replication in chronic lymphocytic leukemia patients. Cancer Genet Cytogenet.

[B9] Sun Y, Wyatt RT, Bigley A, Krontiris TG (2001). Expression and replication timing patterns of wildtype and translocated BCL2 genes. Genomics.

[B10] Smith L, Plug A, Thayer M (2001). Delayed replication timing leads to delayed mitotic chromosome condensation and chromosomal instability of chromosome translocations. Proc Natl Acad Sci USA.

[B11] Amiel A, Elis A, Maimon O, Ellis M, Herishano Y, Gaber E, Fejgin MD, Lishner M (2002). Replication status in leukocytes of treated and untreated patients with polycythemia vera and essential thrombocytosis. Cancer Genet Cytogenet.

[B12] Korenstein-Ilan A, Amiel A, Lalezari S, Lishner M, Avivi L (2002). Allele-specific replication associated with aneuploidy in blood cells of patients with hematologic malignancies. Cancer Genet Cytogenet.

[B13] Breger KS, Smith L, Thayer MJ (2005). Engineering translocations with delayed replication: evidence for cis control of chromosome replication timing. Hum Mol Genet.

[B14] Chang BH, Smith L, Huang J, Thayer M (2007). Chromosomes with delayed replication timing lead to checkpoint activation, delayed recruitment of Aurora B and chromosome instability. Oncogene.

[B15] Barbosa AC, Otto PA, Vianna-Morgante AM (2000). Replication timing of homologous alpha-satellite DNA in Roberts syndrome. Chromosome Res.

[B16] Hansen RS, Stoger R, Wijmenga C, Stanek AM, Canfield TK, Luo P, Matarazzo MR, D'Esposito M, Feil R, Gimelli G (2000). Escape from gene silencing in ICF syndrome: evidence for advanced replication time as a major determinant. Hum Mol Genet.

[B17] Hellman A, Rahat A, Scherer SW, Darvasi A, Tsui LC, Kerem B (2000). Replication delay along FRA7H, a common fragile site on human chromosome 7, leads to chromosomal instability. Mol Cell Biol.

[B18] Reish O, Gal R, Gaber E, Sher C, Bistritzer T, Amiel A (2002). Asynchronous replication of biallelically expressed loci: a new phenomenon in Turner syndrome. Genet Med.

[B19] Norio P, Kosiyatrakul S, Yang Q, Guan Z, Brown NM, Thomas S, Riblet R, Schildkraut CL (2005). Progressive activation of DNA replication initiation in large domains of the immunoglobulin heavy chain locus during B cell development. Mol Cell.

[B20] Farkash-Amar S, Lipson D, Polten A, Goren A, Helmstetter C, Yakhini Z, Simon I (2008). Global organization of replication time zones of the mouse genome. Genome Res.

[B21] Watanabe Y, Fujiyama A, Ichiba Y, Hattori M, Yada T, Sakaki Y, Ikemura T (2002). Chromosome-wide assessment of replication timing for human chromosomes 11q and 21q: disease-related genes in timing-switch regions. Hum Mol Genet.

[B22] Watanabe Y, Ikemura T, Sugimura H (2004). Amplicons on human chromosome 11q are located in the early/late-switch regions of replication timing. Genomics.

[B23] MacAlpine DM, Bell SP (2005). A genomic view of eukaryotic DNA replication. Chromosome Res.

[B24] Schubeler D, Scalzo D, Kooperberg C, Van Steensel B, Delrow J, Groudine M (2002). Genome-wide DNA replication profile for Drosophila melanogaster: a link between transcription and replication timing. Nat Genet.

[B25] Woodfine K, Fiegler H, Beare DM, Collins JE, McCann OT, Young BD, Debernardi S, Mott R, Dunham I, Carter NP (2004). Replication timing of the human genome. Hum Mol Genet.

[B26] Karnani N, Taylor C, Malhotra A, Dutta A (2007). Pan-S replication patterns and chromosomal domains defined by genome-tiling arrays of ENCODE genomic areas. Genome Res.

[B27] Birney E, Stamatoyannopoulos JA, Dutta A, Guigo R, Gingeras TR, Margulies EH, Weng Z, Snyder M, Dermitzakis ET, Thurman RE (2007). Identification and analysis of functional elements in 1% of the human genome by the ENCODE pilot project. Nature.

[B28] White EJ, Emanuelsson O, Scalzo D, Royce T, Kosak S, Oakeley EJ, Weissman S, Gerstein M, Groudine M, Snyder M (2004). DNA replication-timing analysis of human chromosome 22 at high resolution and different developmental states. Proc Natl Acad Sci USA.

[B29] Woodfine K, Beare DM, Ichimura K, Debernardi S, Mungall AJ, Fiegler H, Collins VP, Carter NP (2005). Replication Timing of Human Chromosome 6. Cell Cycle.

[B30] MacAlpine DM, Rodriguez HK, Bell SP (2004). Coordination of replication and transcription along a Drosophila chromosome. Genes Dev.

[B31] Doetschman TC, Eistetter H, Katz M, Schmidt W, Kemler R (1985). The in vitro development of blastocyst-derived embryonic stem cell lines: formation of visceral yolk sac, blood islands and myocardium. J Embryol Exp Morphol.

[B32] Yagi T, Tokunaga T, Furuta Y, Nada S, Yoshida M, Tsukada T, Saga Y, Takeda N, Ikawa Y, Aizawa S (1993). A novel ES cell line, TT2, with high germline-differentiating potency. Anal Biochem.

[B33] Ying QL, Stavridis M, Griffiths D, Li M, Smith A (2003). Conversion of embryonic stem cells into neuroectodermal precursors in adherent monoculture. Nat Biotechnol.

[B34] Rathjen J, Haines BP, Hudson KM, Nesci A, Dunn S, Rathjen PD (2002). Directed differentiation of pluripotent cells to neural lineages: homogeneous formation and differentiation of a neurectoderm population. Development.

[B35] Hanna J, Wernig M, Markoulaki S, Sun CW, Meissner A, Cassady JP, Beard C, Brambrink T, Wu LC, Townes TM (2007). Treatment of sickle cell anemia mouse model with iPS cells generated from autologous skin. Science.

